# Severe asthma ILC2s demonstrate enhanced proliferation that is modified by biologics

**DOI:** 10.1111/resp.14506

**Published:** 2023-04-28

**Authors:** Bilal Malik, Nathan W. Bartlett, John W. Upham, Kristy S. Nichol, John Harrington, Peter A. B. Wark

**Affiliations:** ^1^ Immune Health Program, Hunter Medical Research Institute University of Newcastle Callaghan New South Wales Australia; ^2^ Department of Respiratory Medicine Princess Alexandra Hospital Brisbane Queensland Australia; ^3^ Department of Respiratory and Sleep Medicine John Hunter Hospital New Lambton Heights New South Wales Australia

**Keywords:** ILC2, mepolizumab, omalizumab, proliferation, severe asthma, thymic stromal lymphopoietin receptor, type 2 cytokine, type 2 innate lymphoid cell

## Abstract

**Background and Objective:**

Type 2 (T2) innate lymphoid cells (ILC2s) contribute to airway inflammation and disease in asthma. We hypothesize that ILC2s isolated from people with severe allergic and eosinophilic asthma would exhibit an enhanced T2 inflammatory activity that would be altered following treatment with mepolizumab and omalizumab. We compare peripheral blood (PB) isolated ILC2's proliferative capacity, IL‐5 and IL‐13 secretion and phenotype between healthy without asthma (HC), non‐asthma allergic (NAA), mild asthma (MA) and severe allergic and eosinophilic asthma (SA) subjects. We then determined the impact of 6 months treatment with either mepolizumab or omalizumab on ILC2s physiology of SA subjects.

**Methods:**

ILC2s were sorted and cultured in the presence of IL‐2, IL‐25, IL‐33 and thymic stromal lymphopoietin (TSLP) for 14 days. ILC2s proliferation, phenotypes and functions were assessed using flowcytometry. The ILC2s response was then reassessed following clinically successful treatment of SA subjects with mepolizumab and omalizumab.

**Results:**

SA ILC2s demonstrated increased proliferative capacity, TSLP receptor (TSLPR), GATA3 and NFATc1 protein expressions and increased IL‐5 and IL‐13 release. ILC2s were also capable of releasing IL‐6 in response to stimulation. Mepolizumab treatment reduced ILC2s proliferative capacity and expression of TSLPR, GATA3 and NFATc1. Both mepolizumab and omalizumab were associated with reduced ILC2s release of IL‐5 and IL‐13, only mepolizumab reduced IL‐6.

**Conclusion:**

ILC2s from severe allergic and eosinophilic asthma demonstrated an active phenotype typified by increased proliferation, TSLPR, GATA3 and NFATc1 expression and increased IL‐5, IL‐13 and IL‐6 release. Mepolizumab reduced markers of ILC2s activation.

## Graphical abstract

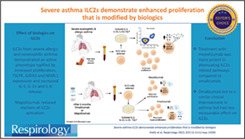

Type 2 innate lymphoid cells (ILC2) from severe eosinophilic asthma subjects demonstrate increased proliferation and type 2 cytokine secretion that was reduced by mepolizumab, and to a lesser extent by omalizumab.

**See related** editorial

## INTRODUCTION

Severe uncontrolled asthma results in a high symptom burden for the individual and disproportionate cost to the health system.^1^ Severe asthma, associated with type 2 high inflammatory disease, is characterized by elevated peripheral blood eosinophils, serum IgE or fractional exhaled nitric oxide (FeNO), and is relatively refractory to treatment with high dose inhaled corticosteroids (ICS).^2^ Treatments that specifically target type 2 immune pathways with biologic monoclonal antibody therapies such as mepolizumab and omalizumab have been shown to reduce exacerbations, systemic corticosteroid usage and improve asthma related quality of life and symptoms.^3^, ^4^, ^5^, ^6^, ^7^

The mechanisms responsible for type 2 immune activation in asthma refractory to ICS remain unclear. Group 2 innate lymphoid cells (ILC2s) are emerging as master regulators of type 2 inflammation and it is becoming increasingly evident that they play an important role in severe eosinophilic asthma and resistance to corticosteroid treatment.^8^, ^9^ In asthma the inflamed airway epithelium secretes IL‐33, IL‐25 and TSLP that attracts and activates ILC2s.^10^, ^11^ Increased numbers of ILC2s have been reported in the airways and sputum of those with severe eosinophilic asthma^9^, ^12^, ^13^ and these ILC2s are an important source of type 2 inflammatory cytokines such as IL‐5 and IL‐13.^13^, ^14^ While blood ILC2s from people with asthma were found to be sensitive to corticosteroids, those from the airways demonstrate resistance to the inhibitory effects of corticosteroids, that was induced through exposure to TSLP and IL‐7.^15^, ^16^ Recently, TSLP along with IL‐33 was also shown to induce the conversion of CD45RA^+^ ILC2s to CD45RO^+^ ILC2s that impart corticosteroid resistance through genes that enhance corticosteroid metabolism.^17^ Therefore, ILC2s under the influence of TSLP released from the airway epithelium are likely to play a crucial role in corticosteroid refractory type 2 airway inflammation that drives severe asthma. Mepolizumab may have a more direct effect on ILC2s as a single dose can markedly reduce serum TSLP and IL‐25 in patients with eosinophilic asthma.^18^ Omalizumab exerts its effects by binding to IgE and preventing its interaction with FcϵRI and FcϵRII receptor that is present on basophils, mast cells, CD4 T‐cells, B‐cells, eosinophils and plasmacytoid dendritic cells that play a key role in allergic inflammation.^19^, ^20^ It might exert an indirect effect on ILC2s as T_H_2 cells influence ILC2s expansion and recruitment to the lungs.^21^

We set out to characterize the functional response of circulating ILC2s from severe allergic and eosinophilic asthma (SA) subjects, comparing these responses to ILC2s from healthy control (HC) subjects, non‐asthma allergic (NAA) subjects and mild asthma (MA) subjects. We then assessed the impact on ILC2s responses in those with SA following 6 months treatment with mepolizumab or omalizumab.

## METHODS

### Subjects

All subjects were 18 years or older, non‐smokers or ex‐smokers with less than 5 pack years smoked. Any subject demonstrating acute respiratory or any other illness within the previous 6 weeks was excluded. We defined allergy as positive skin prick test or radioallergosorbent test (RAST) to aeroallergens such as house dust mite, mixed grass pollen, animal dander, cockroach mix, aspergillus and mould mix. Further details of the subjects and clinical procedures is in Appendix S1 in the Supporting Information.

### Isolation and culture of blood ILC2s

ILC2s isolation was performed on the day of blood collection. Briefly, we cultured blood isolated ILC2s for 14 days and determined proliferation, surface receptor expression and expression of type 2 immune regulating transcription factors GATA3 and NFATc1 (details in Appendix S1 in the Supporting Information).

## RESULTS

### SA ILC2s demonstrate increased TSLPR expression and proliferation post stimulation

**FIGURE 1:**
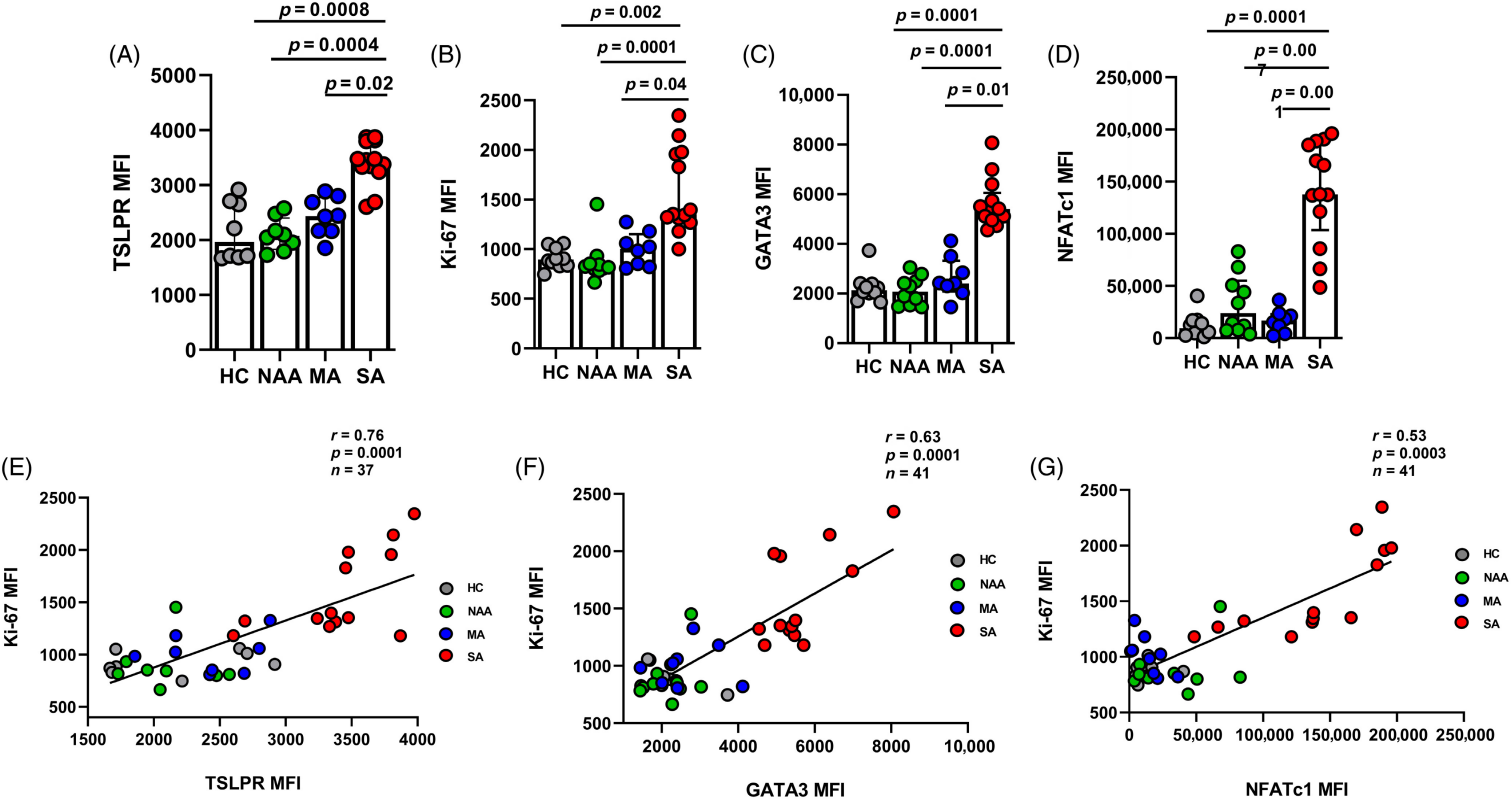
At 14‐day post stimulation ILC2s from SA subjects demonstrated an active phenotype. SA ILC2s demonstrated increased expression of (A) TSLPR, (HC *n* = 8, NAA *n* = 8, MA *n* = 8, SA *n* = 13) (B) Ki‐67, (C) GATA3 and (D) NFATc1 (HC *n* = 10, NAA *n* = 10, MA *n* = 8, SA *n* = 13) compared to subjects from HC, NAA and MA groups post stimulation. ILC2s proliferation correlated positively with the expression of (E) TSLPR, (F) GATA3 and (G) NFATc1. Data are summarized as the median with interquartile range and significance was using Kruskal–Wallis test followed by Dunn's multiple comparison test. For correlation analysis, Spearman correlation test was performed. Two subjects from HC and NAA groups we did not have enough ILC2s post stimulation to analyse extracellular protein expressions. At baseline, we do not have ILC2s post stimulation data of extracellular and intracellular protein expressions from 5 out of 18 SA subjects as at their time of recruitment we were not measuring protein expressions. *p* ≤ 0.05. Healthy without asthma (HC), non‐asthma allergic (NAA), mild asthma (MA) and severe allergic and eosinophilic asthma (SA), type 2 innate lymphoid cells (ILC2s), median fluorescent intensity (MFI), marker of proliferation (Ki‐67), GATA binding protein 3 (GATA3), nuclear factor of activated T‐cells, cytoplasmic 1 (NFATc1) and thymic stromal lymphopoietin receptor (TSLPR).

We found no differences in ILCs numbers and surface receptors in blood between the groups (Figure S1, Appendix S2 in the Supporting Information). Next, we wanted to determine the expression of ILC2s extracellular proteins following 14‐day of stimulation and compare between the groups. We stained cultured ILC2s with antibodies against CD117, CD127, CRTH2, ICOS, ST2, IL‐17RB and TSLPR (Table S1 in the Supporting Information). By flowcytometry (Figure S2A in the Supporting Information) we found that only TSLPR expression was higher in ILC2s from SA subjects compared to the subjects from all other groups (Figure 1A). As TSLP signals through its heterodimeric receptor complex consisting of IL‐7Rα (CD127) and the TSLP receptor subunit (TSLPR), there was no difference in the expression of CD127 between our groups (Figure S2B in the Supporting Information). Furthermore, post stimulation we analysed the expression of Ki‐67 in ILC2s by flowcytometry (Figure S3A in the Supporting Information). ILC2s from SA subjects demonstrated a marked increase in proliferative capacity with increased Ki‐67 expression compared to subjects from all other groups (Figure 1B **)**. ILC2s from SA subjects also demonstrated increased GATA3 (Figure 1C **)** and NFATc1 (Figure 1D **)** expression compared to all other groups. There was no difference in the expression of STAT5 and NF‐KB between the groups post stimulation (data not shown). Moreover, we also found that Ki‐67 expression correlated positively with the expressions of TSLPR (*r* = 0.76, *p* = <0.0001), GATA3 (*r* = 0.63, *p* = <0.0001) and NFATc1 (*r* = 0.53, *p* = <0.0001; Figure 1E–G).

### Increased IL‐5, IL‐13 and IL‐6 secretion by SA ILC2s post stimulation

**FIGURE 2:**
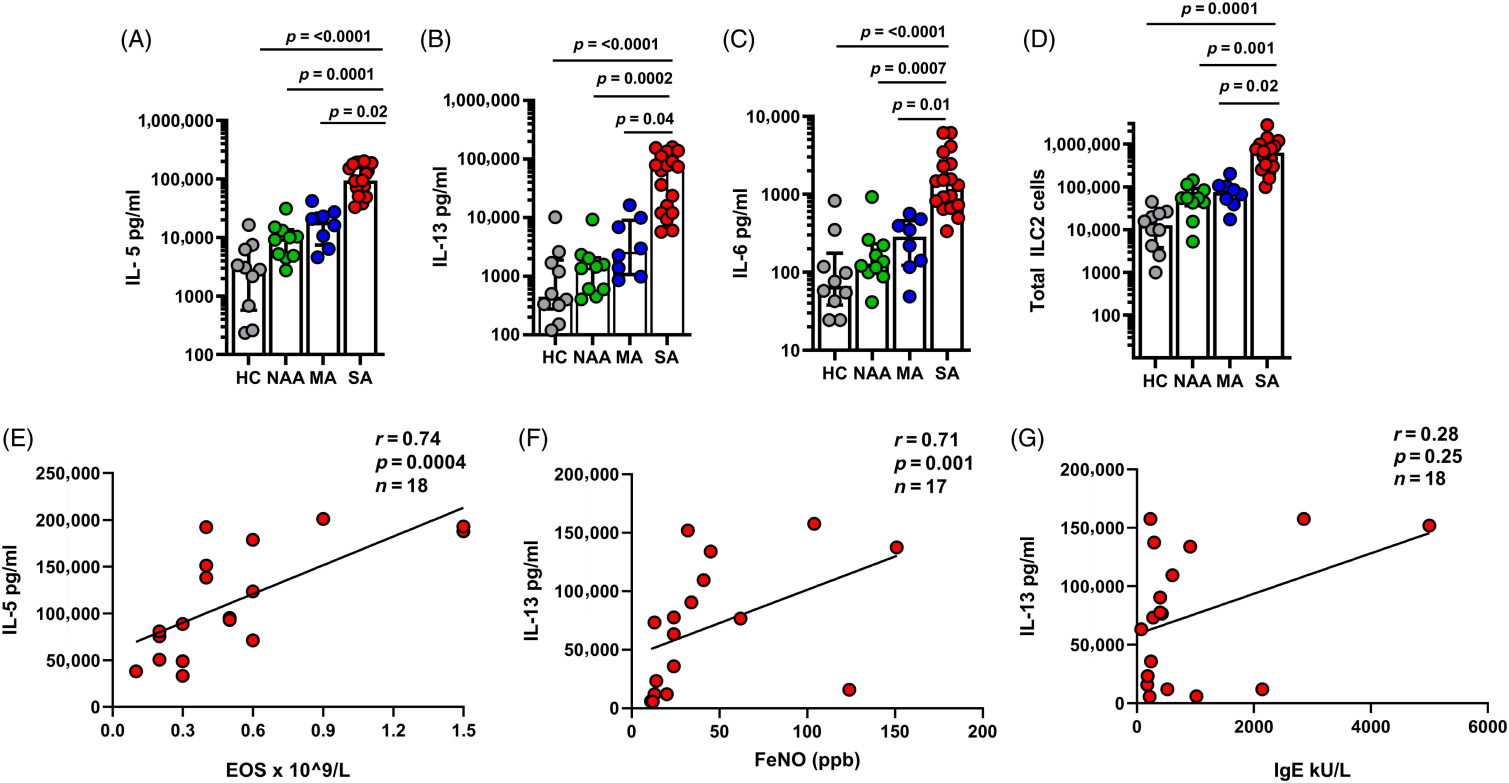
SA ILC2s demonstrates increased IL‐5, IL‐13 and IL‐6 secretion post stimulation. At day 14‐post stimulation, ILC2s from SA group secreted more (A) IL‐5, (B) IL‐13 and (C) IL‐6 compared to HC, NAA and MA groups. Supernatants were analysed for type 1 and type 2 cytokine secretion using Legendplex T_H_1/T_H_2 multi‐analyte flow assay (Biolegend, human T_H_1/T_H_2 panel). (D) Total ILC2s cell count at 14‐day post stimulation. SA subjects demonstrated more total cells compared to HC, NAA and MA groups. Data are shown as median with interquartile range and significance was using Kruskal–Wallis test followed by Dunn's multiple comparison test. IL‐5 and IL‐13 secretion by ILC2s at 14‐day post stimulation correlated positively with SA subjects (E) blood eosinophils and (F) FeNO, respectively. One subject in the SA group was unable to produce recordable FeNO. There was weak correlation of IL‐13 with (G) serum IgE levels. For correlation analysis, Spearman correlation test was performed. HC *n* = 10, NAA *n* = 10, MA *n* = 8, SA *n* = 18. *p* ≤ 0.05. Healthy without asthma (HC), non‐asthma allergic (NAA), mild asthma (MA) and severe allergic and eosinophilic asthma (SA), type 2 innate lymphoid cells (ILC2s), fractional nitric oxide (FeNO) and immunoglobin E (IgE).

Next, we assessed ILC2s release of cytokines post stimulation. We found that SA subjects ILC2s released higher amounts of IL‐5, IL‐13 and IL‐6 compared to ILC2s from the subjects of all other groups (Figure 2A–C). The total ILC2s cell count number post stimulation was greater in SA subjects compared to the other groups (Figure 2D).

We then investigated the association between cultured SA subjects ILC2s secretion of IL‐5 and IL‐13 and clinical evidence of type 2 inflammation in those with SA, as measured by blood eosinophils, serum IgE and fractional exhaled nitric oxide (FeNO). There was a strong positive correlation of SA subjects ILC2s secretion of IL‐5 with blood eosinophils (*r* = 0.80, *p* = <0.0001; Figure 2E) and to a lesser extent, IL‐13 with FeNO (*r* = 0.48, *p* = 0.04; Figure 2F). There was no strong association between IL‐13 secretion and total serum IgE (*r* = 0.30, *p* = 0.22; Figure 2G; Table 1).

**TABLE 1:** Subject characteristics.

Baseline characteristics	HC (*n* = 10)	NAA (*n* = 10)	MA (*n* = 8)	SA (*n* = 18)	*p* value
Age (year)	62 (29, 74)	42 (38, 68)	55 (43,67)	52 (25,72)	0.73
Sex (M/F) (*n*)	6/4	5/5	4/4	10/8	0.14
BMI (kg/cm^2^)	28.53 ± 6.8	26.82 ± 6.5	28.37 ± 6.0	27.42 ± 5.2	0.21
Blood EOS (10^9^/L)	0.10 (0.10, 0.20)	0.10 (0.10,0.30)	0.20 (0.12, 0.35)	0.40 (0.20, 0.74)^a^ ^,^ ^b^	0.0004
Total IgE kU/L	18.50 (11,99)	216 (181. 316)	98.50 (41.325)	413 (236, 1109)^a^ ^,^ ^c^	<0.0001
FEV_1_% predicted	100 (81, 105)	99 (87, 107)	(*n* = 5) 95 (87, 101)	70 (58, 87)^a^ ^,^ ^b^	0.002
FEV_1_/FVC (%)	83 (73, 89)	82 (79, 90)	(*n* = 5) 73 (71, 83)	58 (52,72)^a^ ^,^ ^b^	<0.0001
FeNO (ppb)	N/A	N/A	(*n* = 5) 31 (16, 34)	(*n* = 17) 24 (13, 53)	0.83
ACQ5 score	N/A	N/A	0.30 (0.17, 0.83)	2.9 (2.4, 3.8)	<0.0001
BUD‐equivalent ICS dose (μg/day)	0	0	250 (200, 350)	1816 (1136, 2000)	<0.0001
Maintenance OCS/day (mg)	0	0	0	(*n* = 8/18) 10 (6.25, 13.75)	N/A
Total OCS utilized in previous 6 months (mg)	0	0	0	1255 (990, 1860)	N/A
ILC1/mL blood	39 (34, 87.75)	85 (53.25, 133.5)	101.5 (70.5, 147.3)	84 (32, 151)	0.13
ILC2/mL blood	89 (32.75)	127.5 (72.75, 185.3)	104.5 (77.50, 169)	109 (59, 195)	0.38

*Note*: Data are presented as median (interquartile range IQR) or mean ± SD. We were unable to do lung function tests for three MA subjects due to Covid‐19 related restrictions. One subject in the SA group was unable to produce recordable FeNO. Significance was calculated using one‐way ANOVA followed by Bonferroni multiple comparison test for BMI or Kruskal–Wallis followed by Dunn's comparison test for all other parameters. Mann–Whitney *U* test was performed for FeNO, ACQ‐5 score and ICS.

Abbreviations: ACQ, asthma control questionnaire; BUD, budesonide—BUD‐equivalent ICS dose is calculated as 1 μg of budesonide = 1 μg of beclomethasone = 0.5 μg fluticasone; EOS, eosinophils; HC, heathy subjects; ICS, inhaled corticosteroids; IgE = Immunoglobulin E; ILC, innate lymphoid cells; MA, mild asthma; Median IQR Q1, Q3, first quartiles, third quartiles; N/A, not applicable; NAA, non asthma atopic; OCS, oral corticosteroids; SA, severe allergic eosinophilic asthma.

^a^ *p* ≤ 0.05 compared with HC.

^b^ *p* ≤ 0.05 compared with NAA.

^c^ *p* ≤ 0.05 compared with MA.

### Effect of treatment with mepolizumab and omalizumab on clinical parameters

All subjects were judged by their clinician to have a clinical response and were continued on therapy after 6 months. They demonstrated an improvement in asthma symptoms ACQ5, usage of OCS, number of exacerbations and salbutamol use (Table 2). Subjects treated with mepolizumab also demonstrated decrease in blood eosinophils and FeNO, however neither treatment produced a significant improvement in spirometry (Table 2).

**TABLE 2:** Clinical parameters of SA subjects before and after treatment.

	Mepolizumab			Omalizumab		
Clinical parameters	Pre	Post	*p* value	Pre	Post	*p* value
Subjects (*n*)	10	10		8	8	
Blood EOS × 10^9^/L	0.3 (0.2,0.5)	0.0 (0.0, 0.1)	0.002	0.5 (0.4, 0.9)	0.3 (0.1, 0.4)	0.10
FeNO (ppb)	(*n* = 9) 38 (21, 119)	(*n* = 8) 36 (12, 58)	0.01	24 (13, 39)	16 (8.7, 36)	0.25
ACQ‐5 score	2.8 (2.4, 3.5)	1.2 (0.9, 1.4)	0.003	3.0 (2.4, 4.3)	1.2 (0.8, 2.1)	0.01
FEV1%	62 (54, 91)	(*n* = 8) 78 (50, 100)	0.10	81 (68, 90)	(*n* = 7) 89 (71, 97)	0.21
Total IgE kU/L	964 (228, 2323)	763 (200, 3618)	0.54	354 (232, 420)	1054 (239, 1578)	0.01
Total OCS (mg) use over 6 months prior	1137 (975, 1889)	0.0 (0.0, 758)	0.003	1488 (1078, 1731	512 (0.0, 1449)	0.007
No. of exacerbations 6 months prior	2.0 (1.5, 3.5)	0.0 (0.0, 0.0)	0.003	4.5 (1.0, 6.0)	0.5 (0.0, 2.7)	0.03
Salbutamol inhalation/week	28 (14, 56)	14 (2.0, 14)	0.01	38 (8.7, 56)	7.5 (0.0, 24)	0.01

*Note*: We were unable to do lung function tests for 1 subject in the mepolizumab group post treatment due to Covid‐19 related restrictions. Data are represented as median with IQR and significance was calculated using Wilcoxon matched‐pairs rank test *p* ≤ 0.05.

Abbreviations: ACQ, asthma control questionnaire; EOS, blood eosinophils; FeNo, fractional nitric oxide; FEV_1_, forced expiration volume in 1 second; Median (IQR) Q1, Q3, first quartiles, third quartiles; OCS, oral corticosteroids.

### Mepolizumab treatment impacted ILC2s physiology, omalizumab did not

**FIGURE 3:**
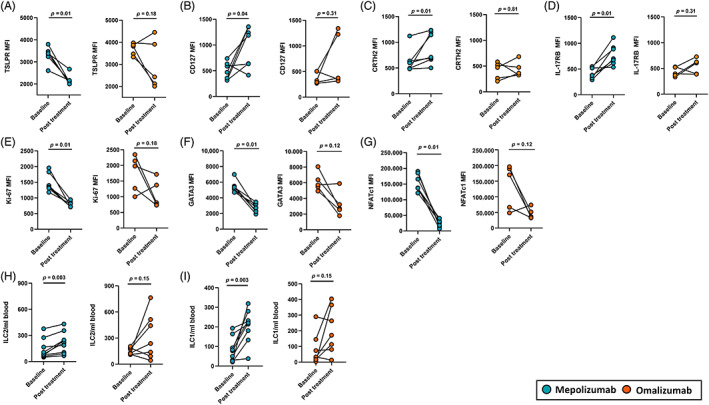
Effect of treatment with mepolizumab and omalizumab on ILC2s physiology. Post treatment, SA subjects treated with mepolizumab demonstrated reduced expression of (A) TSLPR and increased expression of (B) CD127 (C) CRTH2 and (D) IL‐17RB compared to SA subjects treated with omalizumab. Moreover, SA subjects treated with mepolizumab demonstrated reduced expression of (E) Ki‐67, (F) GATA3 and (G) NFATc1 compared to SA subjects treated omalizumab. Mepolizumab *n* = 7, omalizumab *n* = 5. Post treatment in the peripheral blood SA subjects treated with mepolizumab had more (H) ILC2s and (I) ILC1s compared to SA subjects treated with omalizumab. For each subject the type of cells counted were divided by the amount of starting peripheral blood. Mepolizumab *n* = 9, omalizumab *n* = 7. At the 6 months post treatment visit, out of 18 SA subjects, 1 subject in each of mepolizumab and omalizumab group declined blood collection while for 2 subjects in each of mepolizumab group and omalizumab group at their time of recruitment we were not measuring ILC2s extracellular and intracellular protein expressions. Differences between the groups were assessed using Wilcoxon matched‐pairs rank test. *p* ≤ 0.05. Type 1 innate lymphoid cells (ILC1s), type 2 innate lymphoid cells (ILC2s), thymic stromal lymphopoietin receptor (TSLPR). cluster of differentiation 127 (CD127), Chemoattractant receptor‐homologous molecule expressed on T_H_2 cells (CRTH2), Interleukin‐17 receptor B (IL‐17RB), marker of proliferation (Ki‐67), GATA binding protein 3 (GATA3) and nuclear factor of activated T‐cells, cytoplasmic 1 (NFATc1).

We assessed the effect of treatment on SA subjects ILC2s surface receptors at day 14 post stimulation. In SA subjects treated with mepolizumab there was reduced expression of TSLPR and an increase in CD127, CRTH2 and IL‐17RB expression (Figure 3A–D). Treatment with omalizumab had inconsistent effects on the expression of these receptors. (Figure 3A–D). There was no significant difference in the expressions of CD117, ST2 and ICOS before and after treatment with mepolizumab or omalizumab (Figure S4A–C in the Supporting Information). Moreover, ILC2s of subjects treated with mepolizumab exhibited reduced proliferation (Ki‐67), and expression of GATA3 and NFATc1 at day 14 post stimulation (Figure 3E–G). Omalizumab treatment did not result in a reduction in ILC2s Ki‐67, GATA3 and NFATc1 expression (Figure **3**E‐G).Furthermore, peripheral blood ILC2s and ILC1s numbers increased following treatment with mepolizumab, but no effect was seen with omalizumab (Figure 3H,I). In addition, mepolizumab also reduced blood EOS and FeNO compared to omalizumab (Figure S5, Appendix S2 in the Supporting Information).

## DISCUSSION

We have shown that peripheral blood ILC2s from severe allergic and eosinophilic asthma subjects, when stimulated with IL‐2, IL‐25, IL‐33 and TSLP, demonstrate increased proliferative capacity, with increased expression of TSLPR, GATA3 and NFATc1. Treatment with mepolizumab and omalizumab resulted in clinical improvement in all, with reduced exacerbations and improved ACQ‐5 score. In addition, mepolizumab was associated with reduced blood eosinophils and FeNO. Mepolizumab had the greatest impact on ILC2s responses, resulting in reduced ILC2s capacity to proliferate, reduced expression of TSLPR, GATA3 and NFATc1 and an increase in CD127, CRTH2 and IL‐17RB. Both mepolizumab and omalizumab were associated with reduced ILC2s capacity to release IL‐5 and IL13. SA ILC2s also demonstrated a heightened release of IL‐6 that was reduced by mepolizumab.

Type 2 immune activation drives the airway pathology in the majority of children and adults with asthma.^22^ T Helper 2 lymphocytes (T_H_2) cells are not the only source of type 2 cytokines. Experimental animal models of asthma and allergy have determined that ILC2s play an important role in driving type 2 immune responses in asthma.^23^ Chronic inflammation of the airway epithelium in asthma leads to the release of the alarmins IL‐25, IL‐33 and TSLP which is increasingly recognized as an important driver of type 2 airway inflammation^24^ and release of TSLP in particular has been associated with worsened clinical asthma severity^25^ When subjects with severe eosinophilic asthma have been compared to mild asthma and non‐atopic control subjects, greater numbers of CD4 T cells, ILC2s and eosinophil progenitors were found in both blood and sputum, while ILC2s were capable of releasing the greatest amounts of type 2 cytokines, IL‐5 and IL‐13.^9^ Our findings support and expand upon this as we demonstrate that circulating ILC2s in SA subjects exhibit enhanced proliferative capacity associated with TSLPR, GATA3 and NFATc1 expression leading to increased IL‐5 and IL‐13 secretion (Figure 2A,B). The enhanced proliferative capacity of ILC2s appears to be a crucial factor, related to their activation in type 2 inflammation and indicative of their exaggerated immune response. It has been reported that uncontrolled T_H_2 cells proliferation and cytokine secretion was observed in mice that lack *DR6* gene.^26^ The inflammatory signals provided by TSLP with IL‐2 and IL‐33 drive innate lymphoid cells to a ILC2s phenotype and enhances their survival.^27^ Moreover, TSLP also enhances the corticosteroid refractory nature of ILC2s^15^, ^16^ by elevating the expression of *GSTM2* and *MGST2* genes that encode glutathione *S*‐transferase enzyme that metabolize and eliminates corticosteroids.^17^, ^28^

We have shown that post stimulation, ILC2s from SA subjects demonstrate increased expressions of GATA3 and NFATc1 that correlated positively with the expression of the proliferation marker Ki‐67 (Figure 1F,G). This suggests that GATA3 and NFATc1 are involved in controlling ILC2s proliferation and are part of the mechanism controlled by TSLPR. Indeed, this is in accordance with the recent finding where mice deficient of NIP‐45, a NFATc1 interacting protein, had ILC2s with a defect in survival, expressed less GATA3 and lower levels of IL‐5 and IL‐13 compared to controls.^29^ GATA3 has been shown to control the proliferation and survival of CD‐4 T‐cells and ILC2s through the expression of c‐myc, a proto‐oncoprotein.^30^, ^31^ This illustrates a picture in severe allergic and eosinophilic asthma where ILC2s demonstrate enhanced proliferative capacity despite the use of high dose ICS. This may provide a mechanism linking TSLP directly to corticosteroid resistance in ILC2s responses and corticosteroid refractory asthma.

Severe allergic and eosinophilic asthma subjects treatment with mepolizumab and omalizumab resulted in a similar clinical improvement, in asthma symptoms, a reduction in exacerbation frequency, the need to use oral corticosteroids and reduction in salbutamol use.^3^, ^32^ The effect these treatments had on ILC2s responses post stimulation however differed. Mepolizumab treatment had a more consistent effect in reducing ILC2s proliferation, TSLPR, GATA3 and NFATc1 expression and type 2 cytokine secretion compared to omalizumab. Interestingly we also observed that post treatment, the number of circulating blood ILC2s and ILC1s increased, though again this effect was more pronounced with mepolizumab. We propose that reduced airway inflammation resulted in less ILC2s infiltrating the lungs post treatment and hence more of these cells were circulating in the blood. A similar phenomenon but in reverse has been demonstrated, where ILC2s numbers decreased in the blood and increased in bronchial lavage following allergen challenge in mild asthma patients.^33^

Mepolizumab may have a more direct effect on ILC2s as it has been demonstrated that a single dose can markedly reduce serum TSLP and IL‐25 in patients with non‐allergic eosinophilic asthma.^18^ Since it has also been demonstrated that mice lacking TSLP signalling has reduced numbers of ILC2 progenitors in the bone marrow compared to wild type mice,^34^ this might explain how mepolizumab effected the physiology of ILC2s in our study after 6 months of treatment. The direct effect of IL‐5 receptor blockade on reducing airway infiltration on eosinophils may also influence this change in ILC2s biology, as eosinophils attract and activate ILC2s in asthma^35^ Furthermore, it has also been reported that ILC2s are activated by cysteinyl leukotrienes via NFAT signalling^36^ and prostaglandins D2^37^ so depletion of eosinophils by mepolizumab can effect ILC2s activation as eosinophils are shown to secrete cysteinyl leukotrienes^38^ and prostaglandins.^39^ In support of this mepolizumab treatment also appeared to lead to a more consistent reduction in peripheral blood eosinophils and ILC2s secretion of IL‐5 and IL‐13 compared to omalizumab.

Despite the good clinical response seen with omalizumab treatment, it demonstrated a weaker and inconsistent effect on ILC2s responses in terms of proliferation, TSLPR, GATA3 and NFATc1 expression though it did result in a reduced ability of these cells to release IL‐5 and IL‐13 post treatment. This may reflect the heterogenous nature of disease in SA and highlights the importance in dysregulation of both adaptive and innate immune pathways. Omalizumab exerts its effects by binding to IgE and preventing its interaction with FcϵRI and FcϵRII receptor that is present on basophils, mast cells, CD4 T‐cells, B‐cells, eosinophils and plasmacytoid dendritic cells that play a key role in allergic inflammation.^19^, ^20^ Its effect on ILC2s is indirect as T_H_2 cells influence ILC2s expansion and recruitment to the lungs.^21^ Our experience also reflects that seen in a recent prospective observational cohort of omalizumab in severe allergic asthma, where treatment resulted in a good response in terms of reduction in exacerbation frequency and improved asthma symptom control and the greatest effect was seen in those with elevated blood eosinophils and FeNO.^40^

The finding of increased IL‐6 release from SA ILC2s is not typically seen in type 2 immune response. Blood IL‐6 levels has previously been shown to be elevated in a severe asthma cohort and associated with increased exacerbations.^41^ However, we now demonstrate that SA ILC2s are capable of releasing IL‐6 and this is modified in response to mepolizumab. SA ILC2s demonstrated a 100‐fold increased release of IL‐6 following stimulation with IL‐2, IL‐33, IL‐25 and TSLP compared to mild asthma and the controls. Cutaneous ILC2s have previously been shown to release IL‐6 together with IL‐5 in a TLR2/NOD2 dependent fashion to bacteria.^42^ While IL‐6 is usually considered to be triggered in response to type 1 or type 17 immune responses our results demonstrate a more complex picture where ILC2s may contribute to its release and its effects in severe asthma, though the mechanisms of this remain to be defined.

We have demonstrated that ILC2s from subjects with SA, demonstrate an increased proliferative capacity and strong type 2 immune activation, with increased expression of TSLPR, GATA3 and NFATc1 resulting in enhanced cytokine release of IL‐5 and IL‐13. We also demonstrate that these ILC2s in SA are capable of releasing IL‐6 in response to stimulation. Furthermore, our results also demonstrate that treatment with mepolizumab was more potent in attenuating ILC2s related pathways compared to omalizumab. Omalizumab led to a similar clinical improvement in asthma but had less measurable effect on ILC2s.

## AUTHOR CONTRIBUTION

**Bilal Malik:** Conceptualization (lead); data curation (lead); formal analysis (lead); investigation (lead); methodology (lead); validation (lead); visualization (lead); writing – original draft (lead); writing – review and editing (lead). **Nathan W. Bartlett:** Conceptualization (supporting); data curation (supporting); formal analysis (supporting); investigation (supporting); methodology (supporting); project administration (supporting); supervision (equal); validation (supporting); visualization (supporting); writing – original draft (supporting); writing – review and editing (supporting). **John W. Upham:** Formal analysis (supporting); investigation (supporting); methodology (supporting); supervision (equal); writing – review and editing (supporting). **Kristy S. Nichol:** Data curation (supporting); methodology (supporting); project administration (supporting); validation (supporting); visualization (supporting); writing – review and editing (supporting). **John Harrington:** Methodology (supporting); project administration (supporting); visualization (supporting); writing – review and editing (supporting). **Peter A. B. Wark:** Conceptualization (supporting); data curation (supporting); formal analysis (supporting); funding acquisition (lead); investigation (supporting); methodology (supporting); project administration (supporting); resources (lead); supervision (lead); validation (supporting); visualization (supporting); writing – original draft (supporting); writing – review and editing (supporting).

## CONFLICTS OF INTEREST STATEMENT

We thank Prof. Peter Howarth and Ms Daniela Eassey from GSK for their support. The study was also partly supported by independent investigator grants from Glaxo Smith Kline (GSK) to Bilal Malik and Peter A. B. Wark. GSK had no direct input into the design of experiments, interpretation of the results or writing of the manuscript. John W. Upham declares payments from Novartis, Astra Zeneca, GSK and Sanofi for educational events. The payments were made to his institution. John W. Upham also declares being part of advisory board for Astra Zeneca, GSK and Sanofi. He also declares travel support from Sanofi for attending conference. John W. Upham is the president of Thoracic Society of Australia and New Zealand. John Harrington declares honoraria from GSK, Astra Zeneca and Novartis for educational events. He also declares that he was part of GSK advisory board. John Harrington is also Respiratory Nursing Co‐chair of New South Wales Agency for Clinical Innovation. Peter A. B. Wark declares to have received payments and honoraria for unrelated work from GSK, Astra Zeneca, Boehringer Ingelheim, Novartis and Vertex. Peter A. B. Wark is board member of Cystic Fibrosis Australia and National Asthma Council of Australia. The remaining author did not make disclosures.

## HUMAN ETHICS APPROVAL DECLARATION

The study was approved by Hunter New England Research Ethics Committee (2019/ETH12348) and informed consent was obtained from all subjects. Healthy controls (HC), non‐asthma atopic (NAA) and mild asthma (MA) subjects were recruited at Hunter Medical Research Institute while severe allergic eosinophilic asthma (SA) subjects were recruited as part of the ‘Choosebetweenamab’ trial ACTRN12618000850279 at https://www.anzctr.org.au/

## Supporting information

Supporting Information

**Visual Abstract** Severe asthma ILC2s demonstrate enhanced proliferation that is modified by biologics

## Data Availability

Data are available at public repository at http://flowrepository.org/id/FR-FCM-Z65F.
