# Supplementary material for: Severe asthma ILC2s demonstrate enhanced proliferation that is modified by biologics
Source: Respirology. 2023 Apr 28;28(8):758–66. doi: 10.1111/resp.14506 (PMC10946917; doi:10.1111/resp.14506)
Supplement: Supplementary file 1 — Supporting Information [file RESP-28-758-s002.docx]

**SUPPORTING INFORMATION**

**Severe asthma ILC2s demonstrate enhanced proliferation that is modified by biologics**

Bilal Malik PhD^1^, Nathan W. Bartlett PhD^1^, John W. Upham PhD^3^, Kristy S Nichol B. Biomed Sci^1^, John Harrington M.Sc, Peter A.B. Wark PhD^1, 2^.

^1^ Immune Health Program, Hunter Medical Research Institute, University of Newcastle, New South Wales 2305, Australia.

^2^ Department of Respiratory and Sleep Medicine, John Hunter Hospital, New South Wales 2305, Australia.

^3^ Department of Respiratory Medicine, Princess Alexandra Hospital, Queensland 4102, Australia.

**Appendix S1-Supplementary Methods:**

**Subjects**

All SA subjects were recruited through the severe asthma clinic at John Hunter Hospital, NSW, while HC, NAA and MA subjects were recruited at Hunter Medical Research Institute (HMRI). All subjects were non-smokers or ex-smokers with less than 5 pack years smoked. HC and NAA subjects had no history of current or previous asthma and no evidence of airflow obstruction as assessed by spirometry. Asthma was defined by a doctor diagnosis of asthma together with positive bronchodilator response of ≥12% and ≥200 mL of FEV_1_ when compared to the baseline values or airway hyper responsiveness defined by 20% or 15% decline in FEV_1_ during direct or indirect bronchial provocation test respectively or evidence of demonstratable peak flow variability of more than 15% between two highest and two lowest peak expiratory flow for 28 days. MA subjects had asthma that was controlled by GINA step 1 or step 2 of treatment with low dose inhaled corticosteroids (ICS). Severe poorly controlled asthma was defined as; disease that was not controlled despite treatment with high dose ICS (>500mcg of fluticasone or equivalence per day) in combination with a long-acting beta agonist for a period of at least 12 months under the supervision of a specialist respiratory physician (GINA step 5). They also had to demonstrate persistent poor asthma control as measured by an ACQ-5 score of ≥2.0, or requiring treatment with regular oral corticosteroids (OCS) daily for at least 6 weeks or had used a cumulative dose of 500 mg of OCS in preceding year, and they had to have experienced at least 1 severe asthma exacerbation, requiring documented use of systemic corticosteroids (oral corticosteroids initiated or increased for at least 3 days, or parenteral corticosteroids) prescribed/supervised by a physician. In addition, they had to demonstrate a type 2 high disease with persistent eosinophilic and allergic asthma; with a blood eosinophil count of ≥300 cells/ul or ≥150 cells/ul if patients were on systemic corticosteroids and evidence of allergy with an elevated total serum IgE >30IU/ml positive skin prick test or RAST to aeroallergens, performed within the last 12 months. Spirometry was performed according to ATS/ERS guidelines ^1^. FeNO was measured for MA and SA subjects according to ATS standards ^2^. Baseline details of all the subjects are presented in **(Table 1 in the main publication)** and further details of the SA subjects treatment randomization is given in the online supplement.  At the 6 months post treatment visit out of 18 SA subjects, 1 subject in each of mepolizumab and omalizumab group declined blood collection. No participants in the study had acute respiratory tract infections in the preceding four weeks at the time of recruitment.

**Treatment randomisation:**

SA subjects were randomized for treatment with either mepolizumab or omalizumab for the period of 6 months. The subject’s clinical characteristics including ICS & OCS usage, FeNo, blood eosinophils, asthma control questionnaire (ACQ-5), serum IgE, number of exacerbations, FEV_1_ and salbutamol inhalations were recorded and compared. Participants were randomized (1:1) using clinical research design & statistics (CReDITSS) online randomisation engine (CORE), though the investigator and the participant were blinded to receive either Mepolizumab or Omalizumab.

**Human Blood collection , PBMC isolation and ILC enrichment**

Blood was collected for peripheral blood mononuclear cells (PBMCs) isolation, full blood count, total IgE and IgE RAST test. **Peripheral blood was collected into sodium heparin tubes and processed for PBMCs isolation by gradient centrifugation followed by washing twice with PBS containing 10% FCS. Isolated PBMCs were enriched for ILCs using NK cell enrichment kit (Stemcell Technologies) that enriches ILCs and NK cells by positively selecting all the non-NKs cells and non-ILCs ^3^. The enriched cells were further processed for ILC2s sorting on flow cytometer.**

**Flow cytometry:**

**FACS Aria III (BD Biosciences NJ US) flow cytometer was used for ILC2s sorting and protein expression analysis and FlowJo software (BD Biosciences NJ US) was used for all flow cytometer data analysis. Fluorescent minus one and unstained controls were used for the gating thresholds. CS&T beads (BD Biosciences) were used to calibrate flow cytometer every time before running the sample.**

**Peripheral blood ILC2s sorting and surface marker analysis**

**The enriched cells were resuspended into ice cold PBS containing 10% FCS and stained with fluorescent antibodies cocktail described in (Table S1) for ILC2s surface marker analysis and sorting.** Before staining, Fc blocking of the cells was done using 5ul of Fc block for 10min at RT (BD bioscience NJ US) for 1 x10^6 cells. These **cells were then treated with brilliant stain buffer (BD bioscience NJ US) and antibodies described in (Table S1). Cells were incubated on ice for 30mins followed by washing twice with ice cold PBS containing 10% FCS. Viability dye Propidium iodide (PI) was added and cells were again incubated on ice for 15mins before acquiring them on FACS ARIA III (BD Bioscience NJ US). ILC2s were sorted as CD45^+^, lineage^-^ (CD3, CD14, CD16, CD19, CD20, CD56, CD11b, CD11c, CD123), CD127^+^, CD117^+^ and CRTH2^+^ cells (Fig S1A). Moreover, we also analysed the median fluorescent intensity (MFI) of CRTH2, CD127, CD117, ICOS, ST2, IL-17RB, and TSLPR using antibodies described in (Table S1).**

**ILC2s stimulation and proliferation in-vitro**

**ILC2s were sorted at a cell density of 500 cells per well of 96 well round bottom plate and stimulated in the presence of human recombinant cytokines of IL-2 (Peprotech), IL-33 (Peprotech), IL-25 (Peprotech) and TSLP (Peprotech) all at 500ng/ml ^3^ for 14 days using X-vivo 15 media (Lonza Switzerland). 100ul of culture media was removed and topped up with fresh media once a week without further addition of cytokines. At day 14 post stimulation, cells were centrifuged at 200g for 10 mins, supernatants were collected, added to previous media removal and stored at -80^◦^C for type 1/type 2 cytokine analysis while ILC2s were washed twice with ice cold PBS containing 10% FBS. ILC2s were counted using hematocytometer and were divided for extracellular and intracellular staining.** The expanded population of cells were confirmed as ILC2s by flowcytometer, as **ICOS^+^ (99%), CD127^+^ (100%) (Fig S2A) and GATA3^+^ (97%) (Fig S3A).**

**ILC2s extracellular staining post stimulation**

**On day 14 post stimulation, cells were prepared for extracellular staining of ILC2s surface receptors. A**ntibodies for **CD117, CD127, ST2, IL-17RB, TSLPR, ICOS and CRTH2 were used (Table S1). Cells were incubated on ice for 30mins followed by washing and addition of viability dye PI. Cells were acquired on FACS Aria III (BD bioscience NJ US) and gated as shown in (Fig S2A) and MFI for each marker was analysed.**

**ILC2s intracellular staining post stimulation**

**At day 14 post stimulation, cells were treated with PI followed by incubation on ice for 20mins. Live cells were subsequently sorted using FACS Aria III (BD biosciences NJ US). Live sorted cells were washed twice with PBS and immediately fixed/ permeabilized using precooled 70% ethanol for two hours at -20^◦^C. After two hours, ethanol was removed and cells were washed twice with PBS containing 10% FBS. Antibodies for Ki-67, GATA3, NFATc1, STAT5 and NF-KB were used (Table S1) and cells were incubated for 30mins. This was followed by washing twice with ice cold PBS containing 10% FBS and cells were acquired on FACS Aria III (BD biosciences) and gated as shown in (Fig S3A). MFI for each marker was analysed.**

**Legendplex T_H_1/T_H_2 multiplex assay:**

On day 14 post stimulation cells were centrifuged at 200g for 10mins and supernatants were collected. Supernatants were analysed for type 1 and type 2 cytokine secretion using Legendplex T_H_1/T_H_2 multi-analyte flow assay according to the manufacturer’s instructions. (Biolegend, human T_H_1/T_H_2 panel CA US). Briefly, 12.5ul of assay buffer was added to all the reaction wells of 96-well V-bottom plate. This was followed by adding 12.5ul of standards (top and diluted) and 12.5ul of ILC2s supernatants to their prescribed wells. To all the reaction wells we added 12.5ul mix of capture beads for IL-2, IL-4, IL-5, IL-6, IL-10, IL-13, IFN-γ and TNF-α. For IL-5, IL-13 and IL-6, the kit had 1.3pg/ml, 1.4pg/ml and 1.1pg/ml limit of detection respectively. After a series of steps that involved addition of detection antibodies and colour development, the samples were ready to be acquired on FACS Canto (BD bioscience NJ US) and the data was analysed using LEGENDplex^TM^ Data Analysis Software (Biolegend CA US).

**Statistical analysis:**

Statistical software Graphpad prism version 9.3.1 was used for data analysis. The data is represented as mean ± SD if parametric or the median with interquartile range (IQR) if non-parametric. Differences between the two groups were analysed using Mann-Whitney test or for paired data the Wilcoxon matched-pairs rank test. For more than two groups, differences between groups were analysed using Kruskal-Wallis test followed by Dunn’s multiple comparison test.

**Appendix S2-Supplementary Results:**

**No Difference in ILCs number in the blood between the groups:**

Baseline characteristics of all subjects are given in **(Table 1)**. We recruited 46 subjects: HC= 10, NAA= 10, MA= 8 and SA= 18. Eight SA subjects were on maintenance OCS while all were taking high doses of ICS **(Table 1)**. There were no differences seen in the numbers of circulating blood ILC1s and ILC2s between any of the groups **(Table 1 and Fig S1B & C)**. Blood isolated ILC2s demonstrated no differences in the expression of their canonical surface receptors between the groups **(Fig S1D)**. However, TSLPR expression was higher in SA subjects on OCS than in SA subjects not using regular OCS **(Fig S1E)**. There was no difference in blood ILCs numbers and in the expression of other receptors based on OCS (data not shown).

**Treatment reduced ILC2 type 2 cytokine secretion:**

Following treatment with mepolizumab and omalizumab, ILC2s demonstrated reduced secretion of IL-5 and IL-13 at 14 days post stimulation **(Fig S5A & B)**. We also noted that IL-6 release from SA ILC2s statistically significantly decreased following treatment with mepolizumab and decreased though did not reach statistical significance with omalizumab treatment **(Fig S5C).** When represented as a difference between baseline and 6 months, mepolizumab treatment resulted in a median reduction in IL-5: 73945 pg/ml IQR (25363, 73945), compared to omalizumab 99470 pg/ml (42605, 164743) with no difference between the treatments (p=0.35). Mepolizumab resulted in a median reduction in IL-13: 29380 pg/ml (9509, 133946) and omalizumab 33208 pg/ml (6744, 67902) again with no differences seen between treatments (p=0.63**). Similarly, mepolizumab median reduction in** IL-6: 572.3 (179.3, 1683) compared to omalizumab IL-6: 1066 (-227.9, 3527) with no differences between the treatments (p=0.69). In addition, subjects treated with mepolizumab also demonstrated reduced blood EOS and FeNO post treatment compared to subjects treated with omalizumab **(Fig S5D & E).**

**References:**

1. Pellegrino R, Viegi G, Brusasco V, et al. Interpretative strategies for lung function tests. *European Respiratory Journal.* 2005;26(5):948-968.

2. Dweik RA, Boggs PB, Erzurum SC, et al. An official ATS clinical practice guideline: interpretation of exhaled nitric oxide levels (FENO) for clinical applications. *Am J Respir Crit Care Med.* 2011;184(5):602-615.

3. Camelo A, Rosignoli G, Ohne Y, et al. IL-33, IL-25, and TSLP induce a distinct phenotypic and activation profile in human type 2 innate lymphoid cells. *Blood advances.* 2017;1(10):577-589.

**Table S1: Details of flow cytometry antibodies used**

| **Extracellular staining panel** | **Fluorophore** | **Clone** | **Supplier** |
| --- | --- | --- | --- |
| **CD45** | **Alexa Flour-700** | **2D1** | **Biolegend** |
| **CD127** | **BV510** | **A019D5** | **Biolegend** |
| **CD117** | **BV421** | **YB5.B8** | **BD biosciences** |
| **CRTH2** | **PE-Cy-7** | **BM16** | **Biolegend** |
| **ICOS** | **PE-CF594** | **C398.4A** | **BD biosciences** |
| **ST2** | **APC** | **Polyclonal** | **R&D Systems** |
| **IL-17RB** | **PE** | **170220** | **R&D Systems** |
| **TSLPR** | **BV711** | **1F11/TSLPR** | **BD biosciences** |
| **Lineage panel** |  |  |  |
| **CD3** | **FITC** | **SK7** | **BD biosciences** |
| **CD14** | **FITC** | **MφP9** | **BD biosciences** |
| **CD16** | **FITC** | **3G8** | **BD biosciences** |
| **CD19** | **FITC** | **SJ25C1** | **BD biosciences** |
| **CD20** | **FITC** | **L27** | **BD biosciences** |
| **CD56** | **FITC** | **NCAM16.2** | **BD biosciences** |
| **CD11b** | **FITC** | **ICRF44** | **Biolegend** |
| **CD11c** | **FITC** | **3.9** | **Biolegend** |
| **CD123** | **FITC** | **6H6** | **Biolegend** |
| **Intracellular staining panel** |  |  |  |
| **Ki-67** | **FITC** | **20Raj1** | **Life Technologies** |
| **GATA3** | **BV421** | **L50-823** | **BD bioscience** |
| **NFATc1** | **PE** | **7A6** | **Biolegend** |
| **STAT5-pY694** | **PE-Cy7** | **47/Stat5(pY694)** | **BD bioscience** |
| **NF-KB-p65** | **APC** | **14G10A21** | **Biolegend** |

**Cluster of differentiation (CD), chemoattractant receptor homologous with T-helper cells type 2 (CRTH2),** GATA Binding Protein 3 (GATA3), **interleukin 17 receptor B (IL-17-RB), inducible T-cell costimulatory (ICOS),**  marker of proliferation, (Ki-67), Nuclear factor of activated T-cells, cytoplasmic 1 (NFATc1), nuclear factor kappa B (NF-KB), phycoerythrin (PE), phycoerythrin-cyanine7 (PE-Cy7), phycoerythrin-cyanine fluorescent 594 (PE-CF594), suppressor of tumorigenicity (ST2), signal transducer and activator of transcription (STAT) thymic stromal lymphopoietin receptor (TSLPR).

**Figure S1:** In the peripheral blood there was no significant difference in the number of ILC1s and ILC2s between the groups.


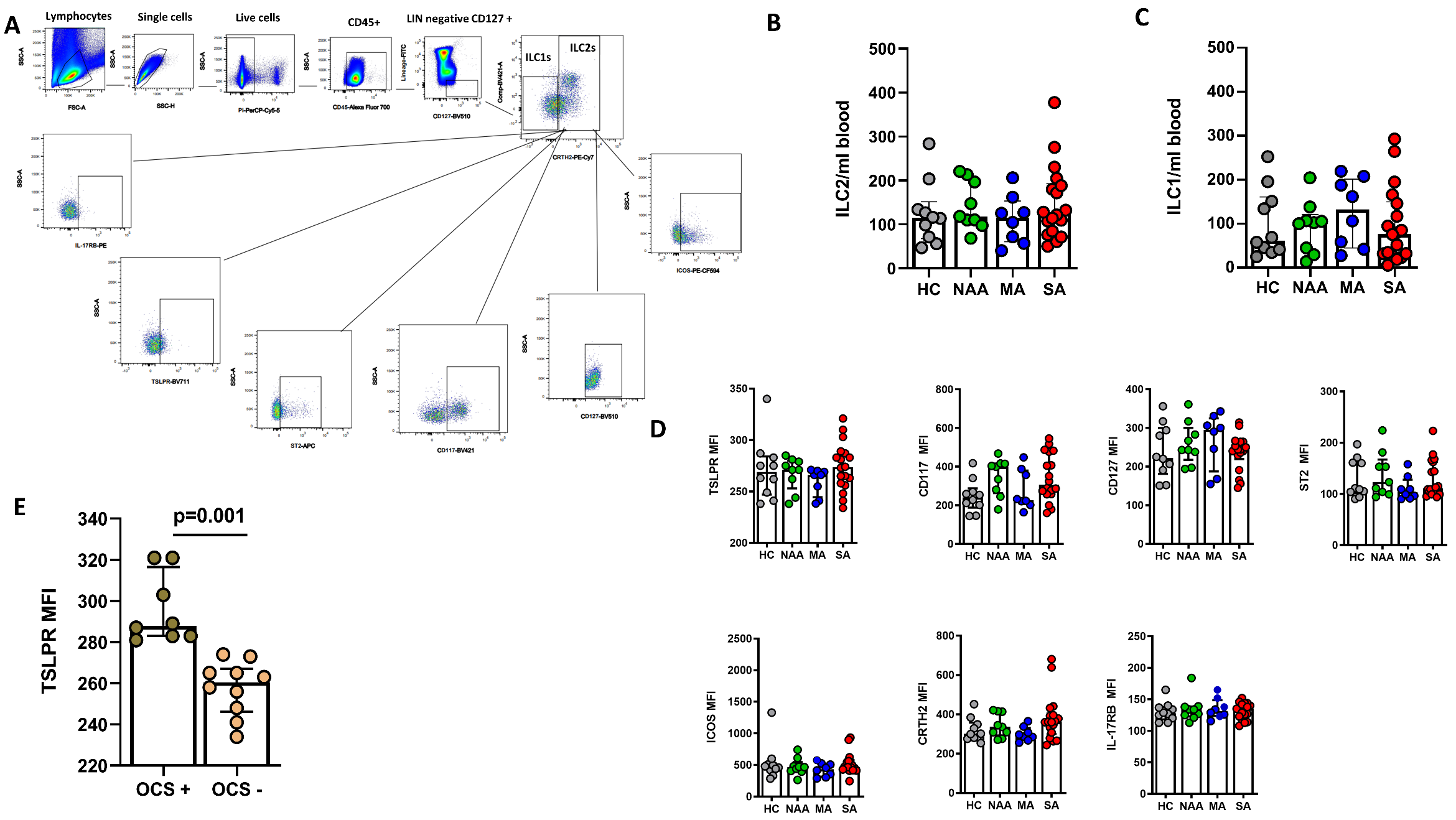


**A)** Gating strategy to isolate and characterize ILC2s from the peripheral blood. There was no difference between **B)** ILC1s and **C)** ILC2s between the groups. For each subject the type of cells counted were divided by the amount of starting peripheral blood. **D)** ILC2s from peripheral blood showed no significant difference in the expression of different surface markers between the groups. **E)** ILC2s cells within the SA group showed difference in TSLPR expression at baseline when characterized based on OCS intake. There was no significant difference in the expression of other markers within SA group (data not shown). Data is summarised as median with interquartile range. **B-D)** Significance was calculated using Kruskal-Wallis test followed by Dunn’s multiple comparison test. **E)** Significance was calculated using Mann-Whitney test. HC n=10, NAA n=10, MA n= 8, SA n=18. p≤0.05. Healthy without asthma (HC), non-asthma allergic (NAA), mild asthma (MA) and severe allergic and eosinophilic asthma (SA), type 2 innate lymphoid cells (ILC2s), type 1 innate lymphoid cells (ILC1s), oral corticosteroids (OCS) median fluorescent intensity (MFI), cluster of differentiation 117 (CD117) cluster of differentiation 127 (CD127), chemoattractant receptor-homologous molecule expressed on T_H_2 cells (CRTH2), forward scatter (FSC), inducible T-cell Costimulator (ICOS), interleukin-17 receptor B (IL-17RB), propidium iodide (PI), suppression of tumorigenicity 2 (ST2), side scatter (SSC), thymic stromal lymphopoietin receptor (TSLPR).

**Figure S2**

**A)** Gating strategy to analyse ILC2s surface receptors 14-day post stimulation.


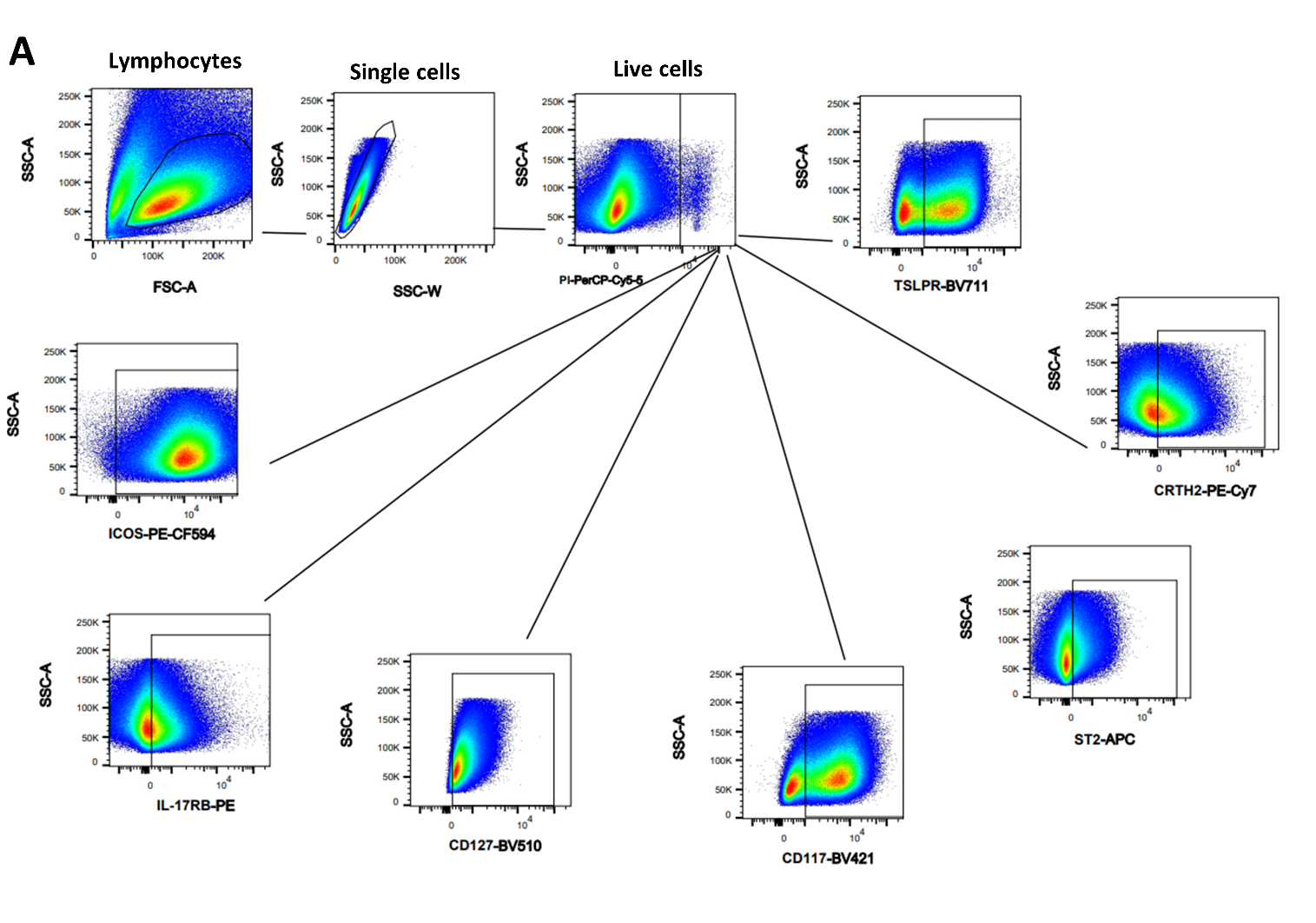


**
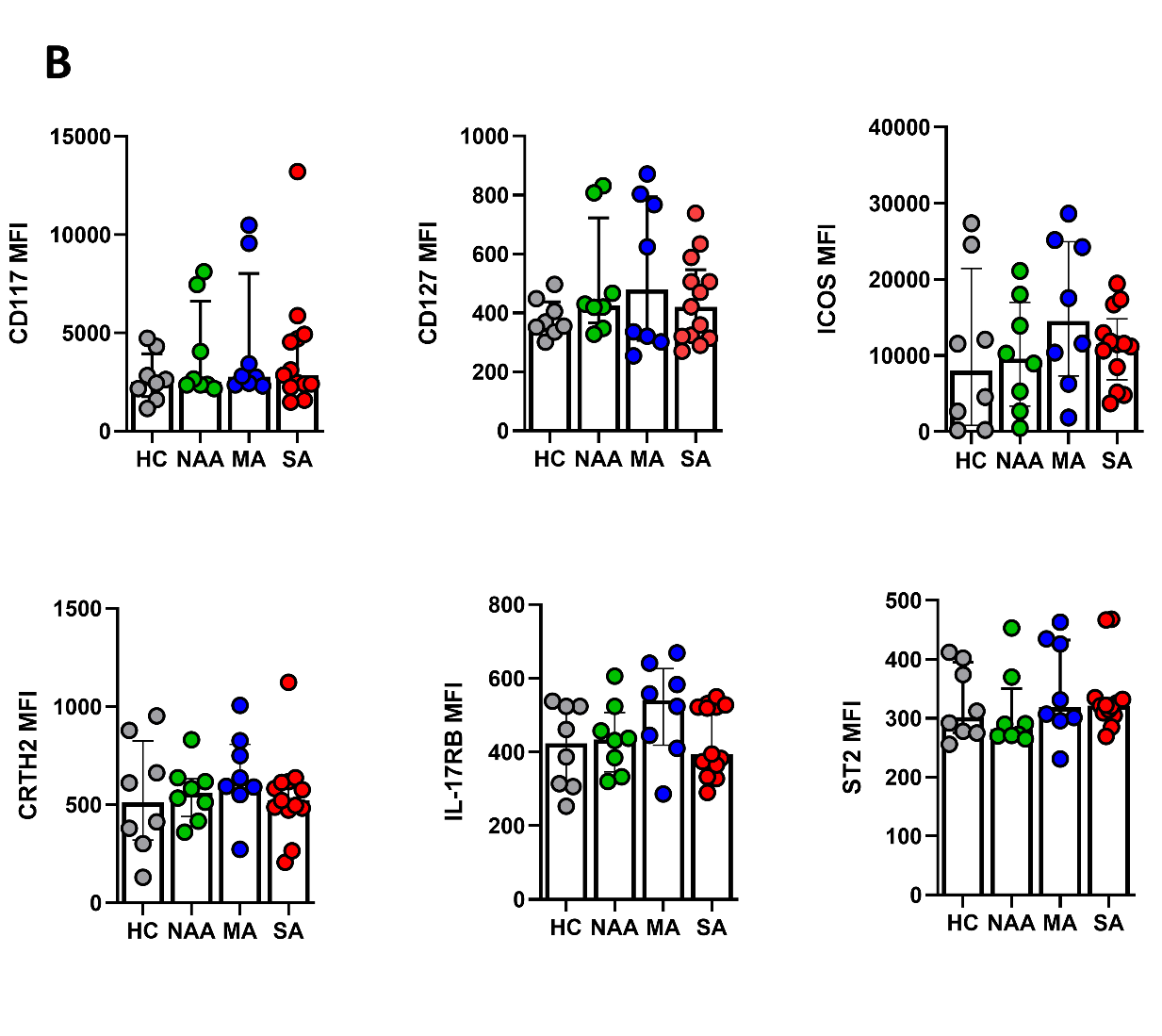
**

**B)** There was no difference in the expression of other ILC2s canonical surface receptors between the groups post stimulation.

HC n=8, NAA n=8, MA n= 8, SA n=13. For 2 subjects from HC and NAA groups we did not have enough ILC2s post stimulation to analyse extracellular protein expressions. At baseline, we do not have ILC2s post stimulation data of extracellular protein expressions from 5 out of 18 SA subjects as at their time of recruitment we were not measuring protein expressions. Data is summarised as the median with interquartile range and significance was using Kruskal-Wallis test followed by Dunn’s multiple comparison test. p≤0.05. Healthy without asthma (HC), non-asthma allergic (NAA), mild asthma (MA) and severe allergic and eosinophilic asthma (SA), type 2 innate lymphoid cells (ILC2s), median fluorescent intensity (MFI), cluster of differentiation 117 (CD117) cluster of differentiation 127 (CD127), chemoattractant receptor-homologous molecule expressed on T_H_2 cells (CRTH2), inducible T-cell Costimulator (ICOS), Interleukin-17 receptor B (IL-17RB), suppression of tumorigenicity 2 (ST2), side scatter (SSC), forward scatter (FSC), propidium iodide (PI).

**Figure S3:** Gating strategy to analyse ILC2s intracellular proteins expression at 14-day post stimulation.


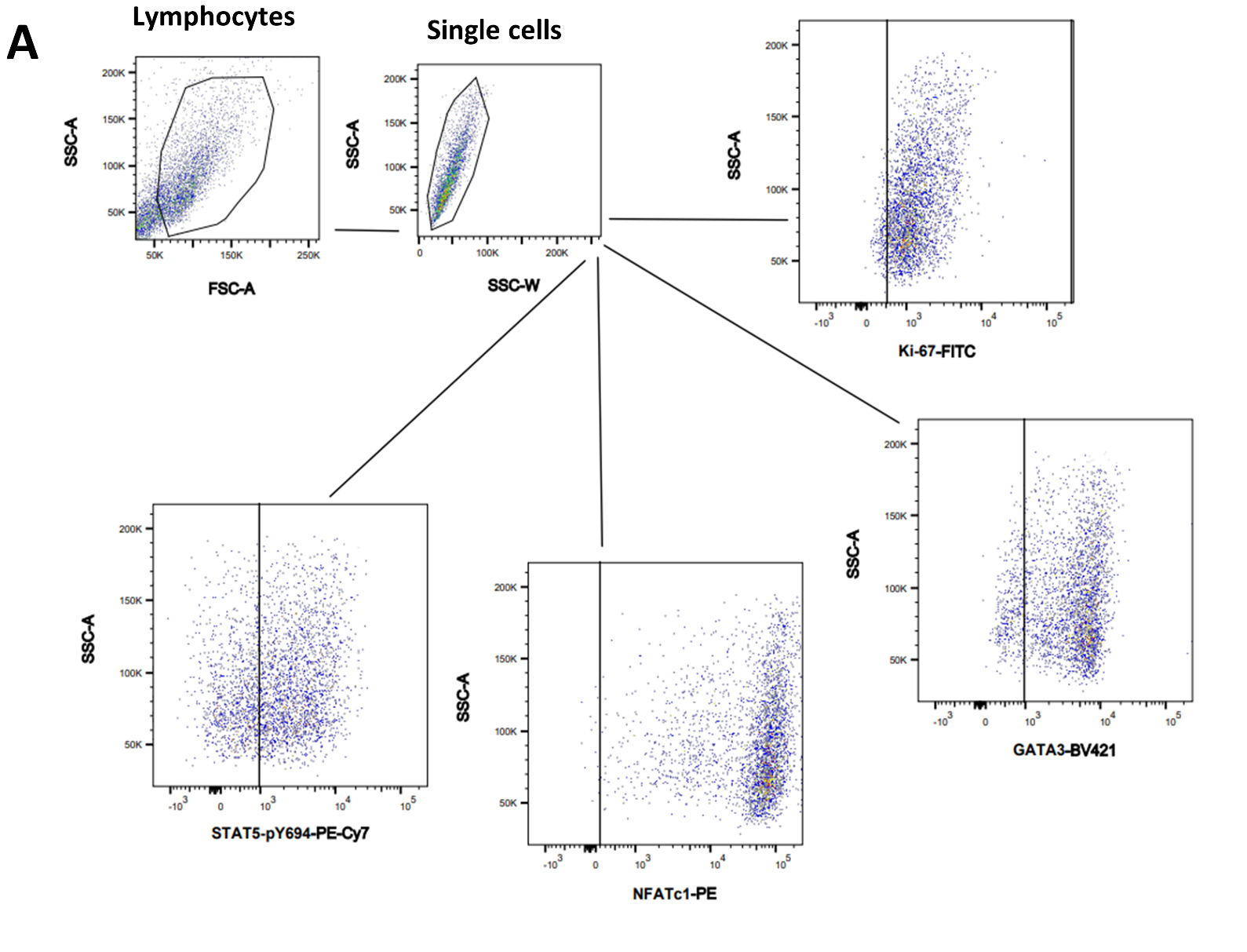


Forward scatter (FSC), marker of proliferation (Ki-67), GATA Binding Protein 3 (GATA3), nuclear factor of activated T-cells, cytoplasmic 1 (NFATc1) side scatter (SSC), signal transducer and activator of transcription (STAT), type 2 innate lymphoid cells (ILC2s).

**Figure S4:** There was no significant difference in the expression **A)** CD117, **B)** ST2 and **C)** ICOS in both mepolizumab and omalizumab groups post treatments.


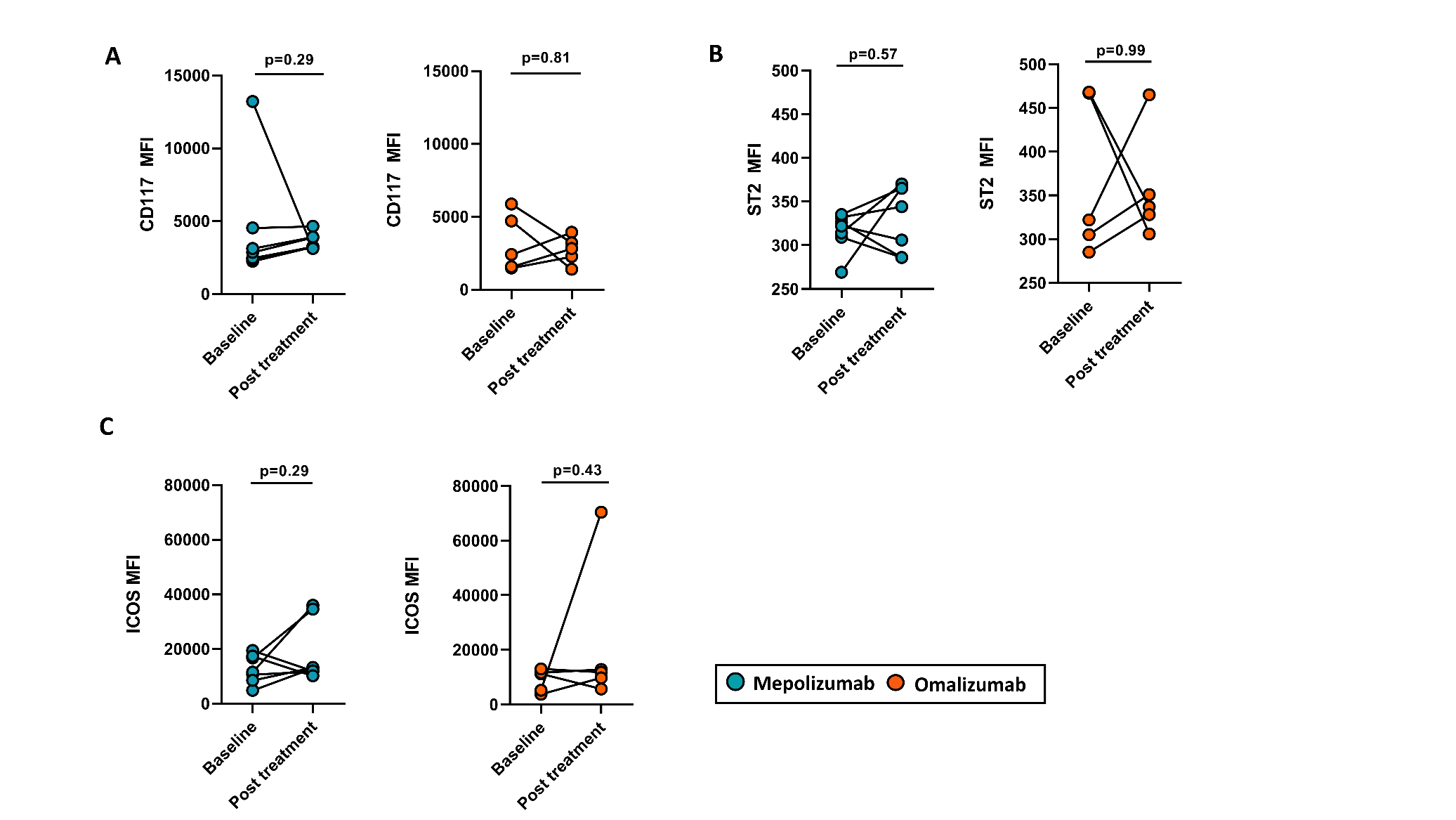


Significance was calculated using Wilcoxon matched-pairs rank test. At the 6 months post treatment visit, out of 18 SA subjects, 1 subject in each of mepolizumab and omalizumab group declined blood collection while for 2 subject in each of mepolizumab group and omalizumab group at their time of recruitment we were not measuring ILC2s extracellular protein expressions. Mepolizumab n=7 and omalizumab n=5. p≤0.05. Cluster of differentiation 117 (CD117), median fluorescent intensity (MFI), suppression of tumorigenicity 2 (ST2), inducible t cell costimulatory (ICOS).

**Figure S5:** Effect of treatment with mepolizumab and omalizumab on ILC2s cytokine secretion, blood eosinophils and FeNO.


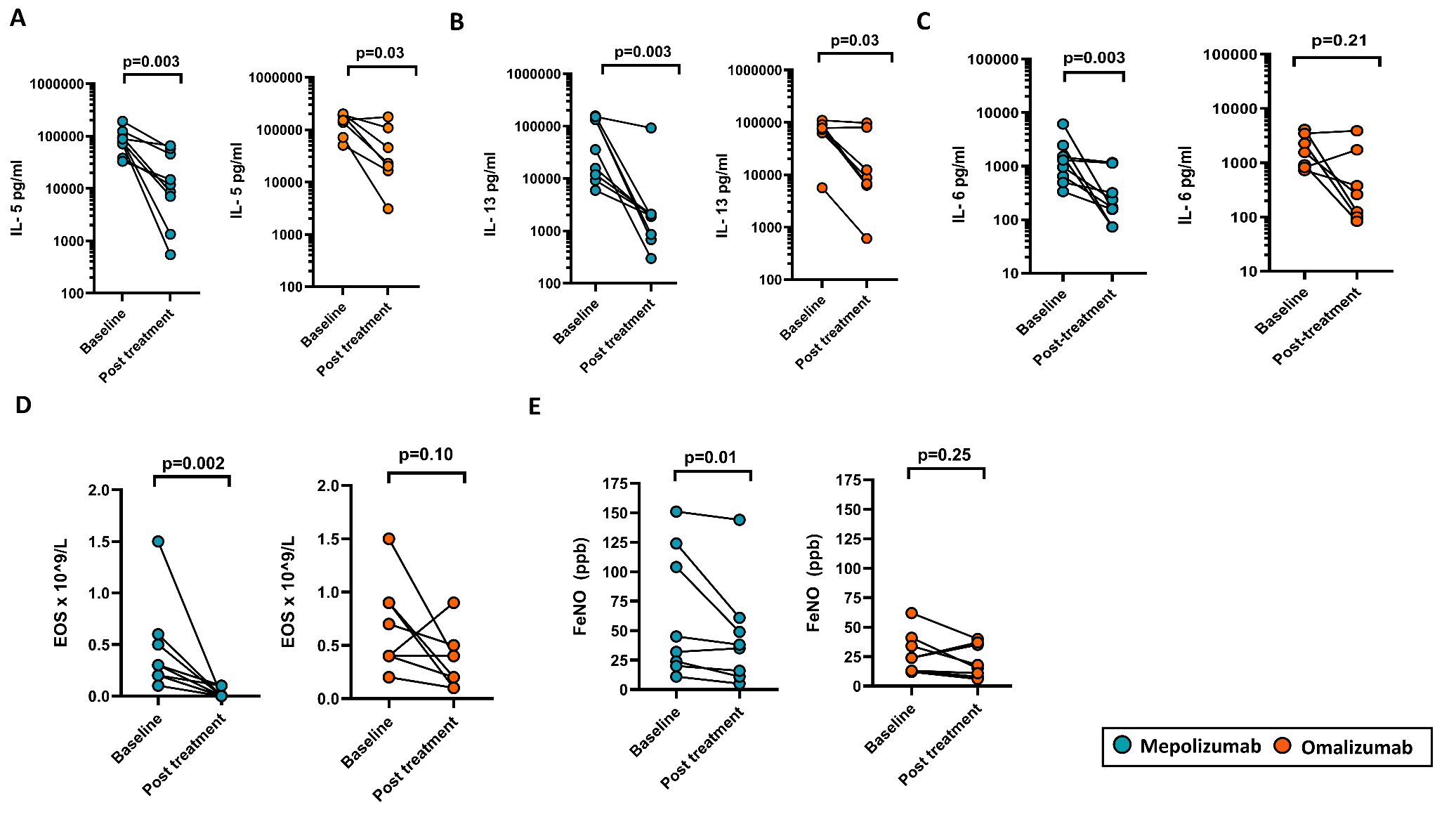


Treatment with mepolizumab and omalizumab reduced ILC2s ability to secrete **F)** IL-5 and **G)** IL-13 while only mepolizumab was able to statistically significantly reduce **H)** IL-6 post treatment. At day 14 post stimulation cell culture media was collected and analysed for IL-5, IL-13 and IL-6 secretion by ILC2s using Legendplex T_H_1/T_H_2 assay. Post treatment, SA subjects treated with mepolizumab demonstrated reduced **I)** EOS numbers and **J)** FeNO compared to SA subjects treated with omalizumab. Significance was calculated using Wilcoxon matched-pairs rank test. At the 6 months post treatment visit, out of 18 SA subjects, 1 subject in each of mepolizumab and omalizumab group declined blood collection. Mepolizumab n= 9 and omalizumab n = 7. p≤0.05. Blood eosinophils (EOS), fractional nitric oxide (FeNO), interleukin (IL).
