# Supplementary material for: Severe asthma ILC2s demonstrate enhanced proliferation that is modified by biologics
Source: Respirology. 2023 Apr 28;28(8):758–66. doi: 10.1111/resp.14506 (PMC10946917; doi:10.1111/resp.14506)
Supplement: Supplementary file 2 — Visual Abstract Severe asthma ILC2s demonstrate enhanced proliferation that is modified by biologics [file RESP-28-758-s001.pdf]

Severe asthma ILC2s demonstrate enhanced proliferation that is modified by biologics

Effect of biologics on ILC2s

ILC2s from severe allergic and eosinophilic asthma demonstrated an active phenotype typified by increased proliferation, TSLPR, GATA3 and NFATc1 expression and increased IL-5, IL-13 and IL-6 release.

Mepolizumab reduced markers of ILC2s activation.

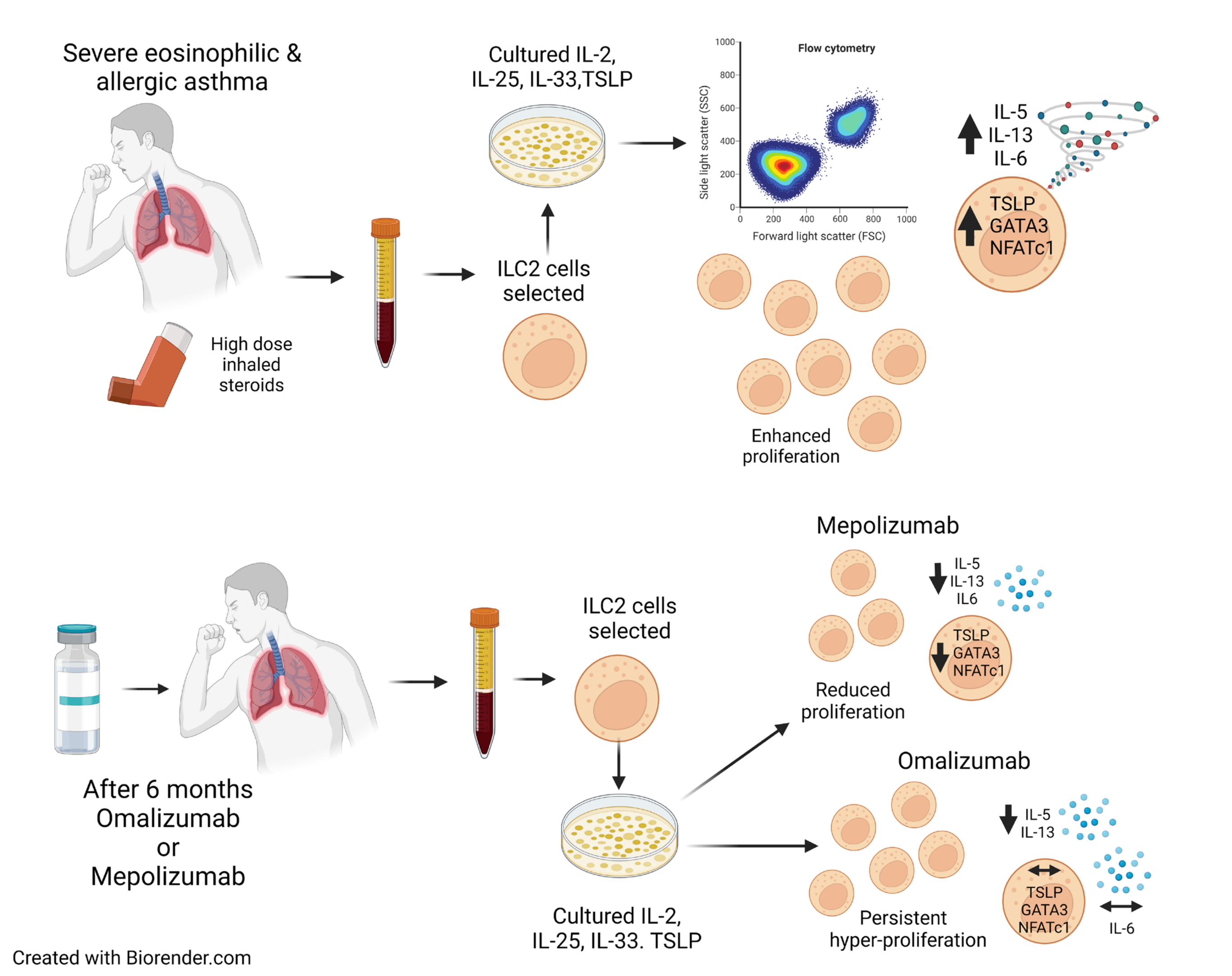

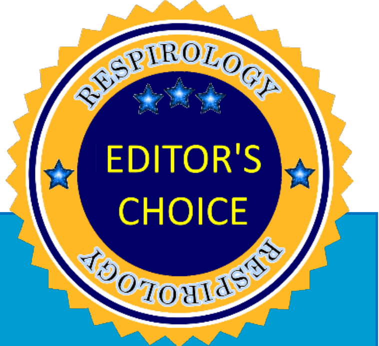

Conclusion

- Treatment with mepolizumab was more potent in attenuating ILC2s related pathways compared to omalizumab.
- Omalizumab led to a similar clinical improvement in asthma but had less measurable effect on ILC2s.

Respirology

Severe asthma ILC2s demonstrate enhanced proliferation that is modified by biologics

Malik et al. Respirology 2023; DOI:10.1111/resp.14506
